# Supplementary material for: PR-SET7 epigenetically restrains uterine interferon response and cell death governing proper postnatal stromal development
Source: Nat Commun. 2024 Jun 10;15:4920. doi: 10.1038/s41467-024-49342-6 (PMC11164956; doi:10.1038/s41467-024-49342-6)
Supplement: Supplementary file 1 — Supplementary Information [file 41467_2024_49342_MOESM1_ESM.pdf]

PR-SET7 epigenetically restrains uterine interferon response and cell death governing proper postnatal stromal development

Haili Bao, Yang Sun, Na Deng, Leilei Zhang, Yuanyuan Jia, Gaizhen Li, Yun Gao, Xinyi Li, Yedong Tang, Han Cai, Jinhua Lu, Haibin Wang, Wenbo Deng, Shuangbo Kong

This file contains:

Supplementary Fig. 1-11

Supplementary Table 1-2

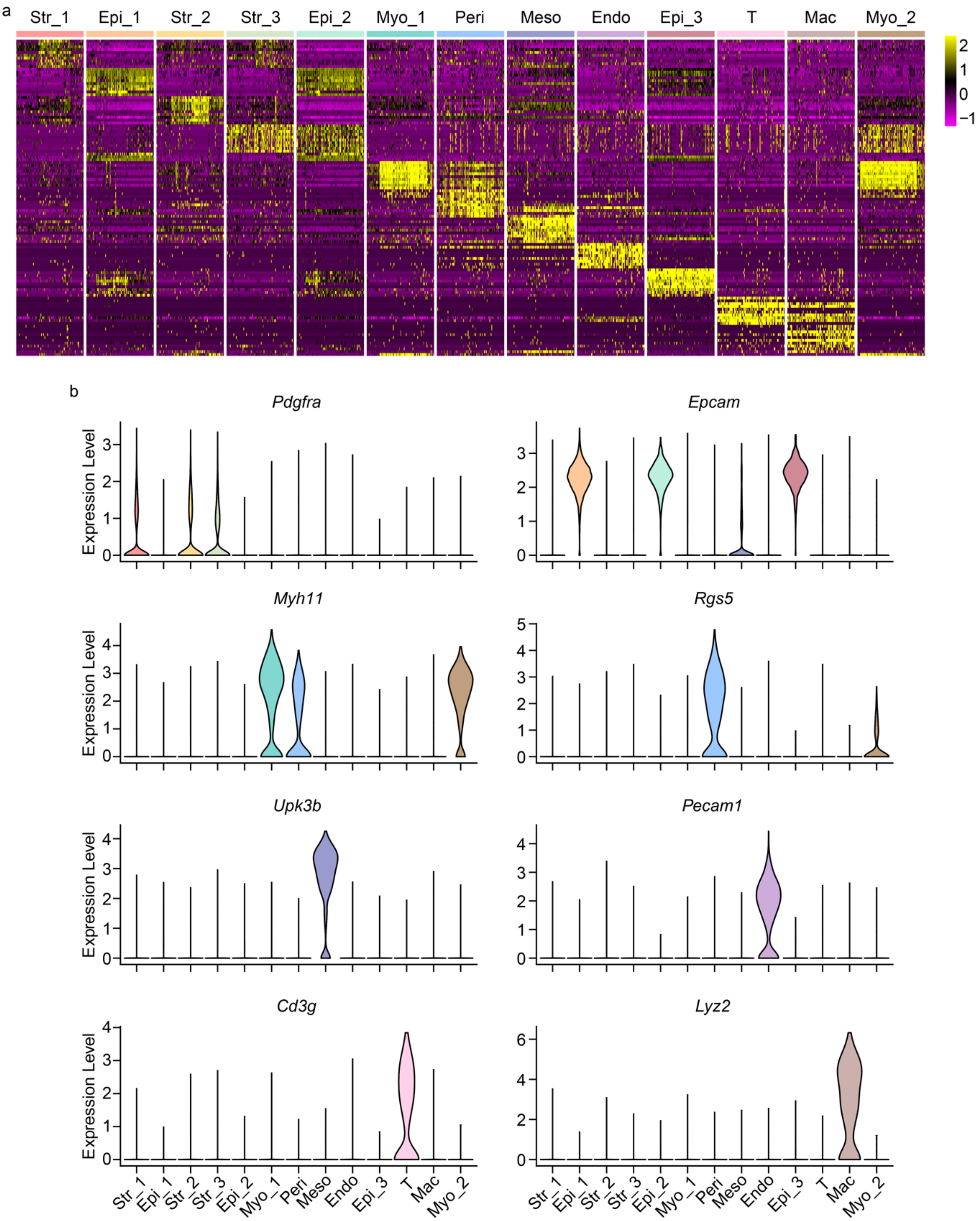
a
Str_1
Epi_1
Str_2
Str_3
Epi_2
Myo_1
Peri
Meso
Endo
Epi_3
T
Mac
Myo_2
2
1
0
−1
b
Pdgfra
Epcam
Myh11
Rgs5
Upk3b
Pecam1
Cd3g
Lyz2
Expression Level

Supplementary Fig. 1 Marker genes of each cell cluster.

a Heatmap showing top ten highly expressed genes in each cell cluster. **b** Violin plot indicating representative marker genes of each cell cluster.

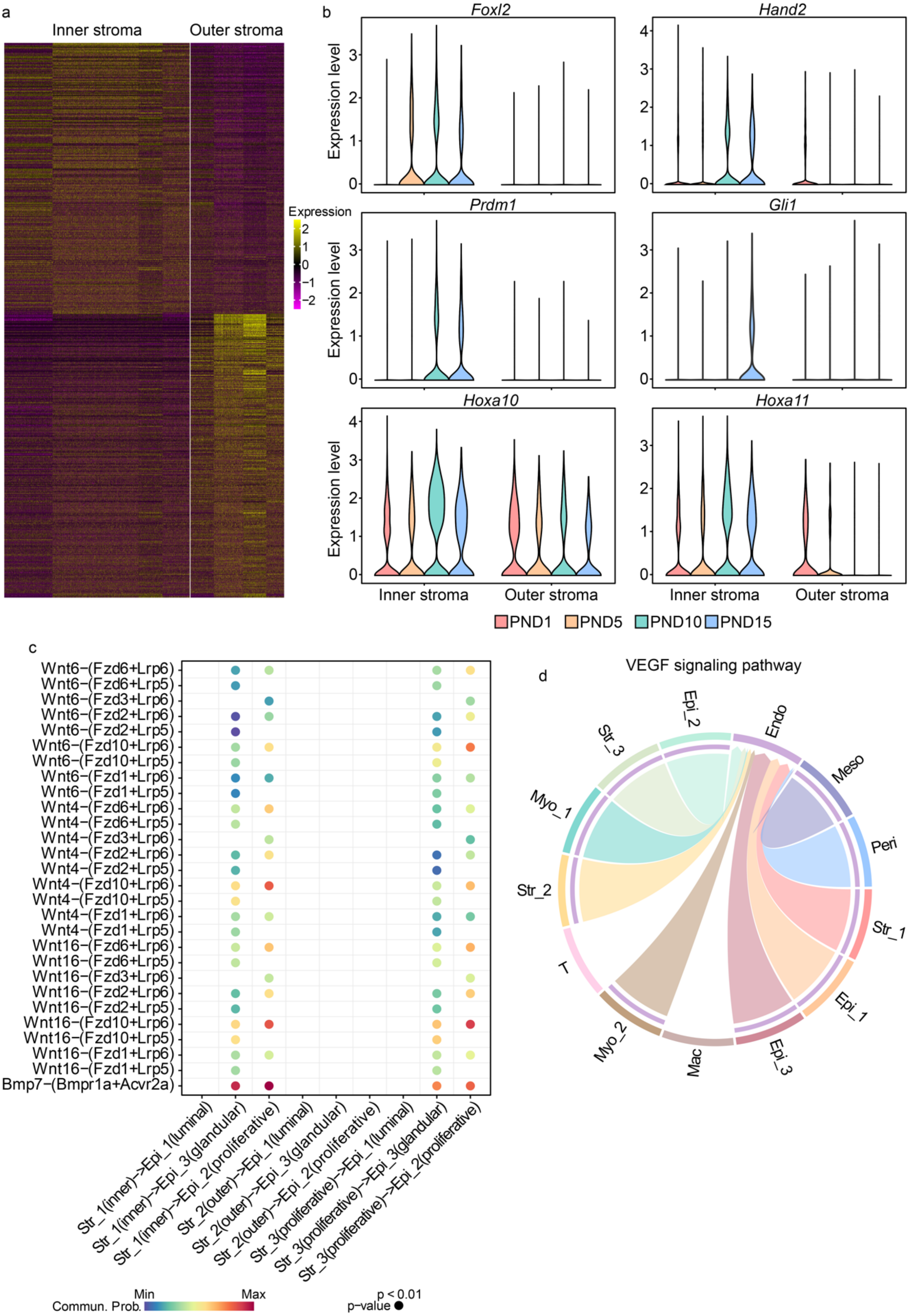
a
Inner stroma
Outer stroma
Expression
2
1
0
−1
−2
b
Foxl2
Hand2
Prdm1
Gli1
Hoxa10
Hoxa11
Expression level
Inner stroma
Outer stroma
PND1
PND5
PND10
PND15
c
Wnt6−(Fzd6+Lrp6)
Wnt6−(Fzd6+Lrp5)
Wnt6−(Fzd3+Lrp6)
Wnt6−(Fzd2+Lrp6)
Wnt6−(Fzd2+Lrp5)
Wnt6−(Fzd10+Lrp6)
Wnt6−(Fzd10+Lrp5)
Wnt6−(Fzd1+Lrp6)
Wnt6−(Fzd1+Lrp5)
Wnt4−(Fzd6+Lrp6)
Wnt4−(Fzd6+Lrp5)
Wnt4−(Fzd3+Lrp6)
Wnt4−(Fzd2+Lrp6)
Wnt4−(Fzd2+Lrp5)
Wnt4−(Fzd10+Lrp6)
Wnt4−(Fzd10+Lrp5)
Wnt4−(Fzd1+Lrp6)
Wnt4−(Fzd1+Lrp5)
Wnt16−(Fzd6+Lrp6)
Wnt16−(Fzd6+Lrp5)
Wnt16−(Fzd3+Lrp6)
Wnt16−(Fzd2+Lrp6)
Wnt16−(Fzd2+Lrp5)
Wnt16−(Fzd10+Lrp6)
Wnt16−(Fzd10+Lrp5)
Wnt16−(Fzd1+Lrp6)
Wnt16−(Fzd1+Lrp5)
Bmp7−(Bmpr1a+Acvr2a)
Str_1(inner)−>Epi_1(luminal)
Str_1(inner)−>Epi_3(glandular)
Str_1(inner)−>Epi_2(proliferative)
Str_2(outer)−>Epi_1(luminal)
Str_2(outer)−>Epi_3(glandular)
Str_2(outer)−>Epi_2(proliferative)
Str_3(proliferative)−>Epi_1(luminal)
Str_3(proliferative)−>Epi_3(glandular)
Str_3(proliferative)−>Epi_2(proliferative)
Commun. Prob.
Min
Max
p-value
p < 0.01
d
VEGF signaling pathway
Epi_2
Endo
Meso
Peri
Str_1
Epi_1
Epi_3
Mac
Myo_2
T
Str_2
Myo_1
Str_3

Supplementary Fig. 2 Distinct features of the inner and outer stromal cells. **a** Heatmap showing gene expression in the inner and outer stromal cells. **b** Violin plot indicating the expressions of *Foxl2*, *Prdm1*, *Hand2*, *Gli1*, *Hoxa10* and *Hoxa11* in the inner and outer stromal cells throughout the postnatal period. **c** CellChat analysis displaying the WNT and BMP signaling pathways from stromal cells to epithelial cells. **d** CellChat analysis displaying the VEGF signaling pathway among different cell clusters.

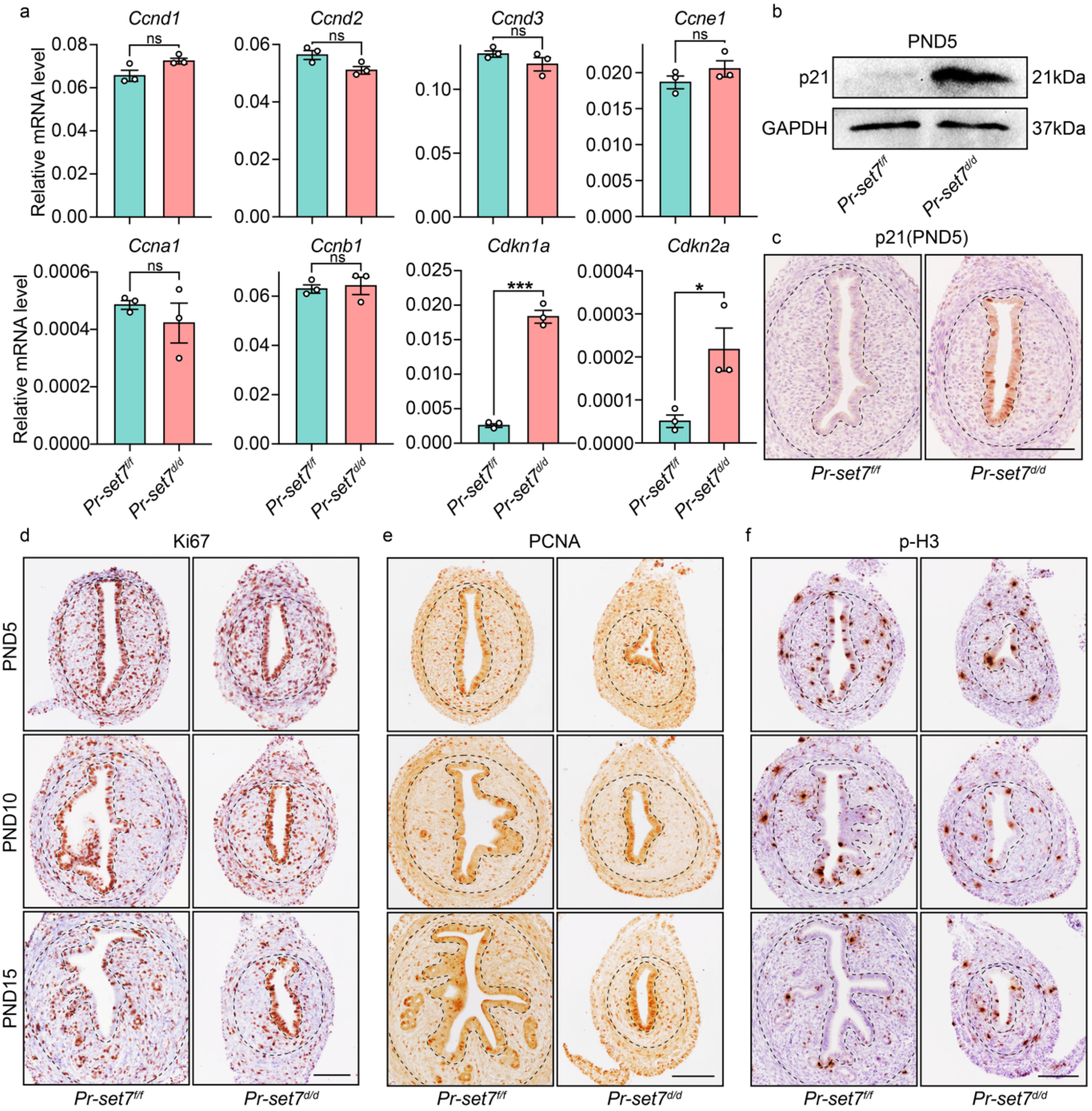

a
Ccnd1
Ccnd2
Ccnd3
Ccne1
Ccna1
Ccnb1
Cdkn1a
Cdkn2a
Relative mRNA level
ns
***
*
Pr-set7f/f
Pr-set7d/d
b
PND5
p21
GAPDH
21kDa
37kDa
c
p21(PND5)
d
Ki67
e
PCNA
f
p-H3
PND5
PND10
PND15

Supplementary Fig. 3 The proliferation of stromal cells was not affected by *Pr-set7* deletion.

a QRT-PCR analysis of the mRNA levels of cyclins and CKIs in *Pr-set7*$^{f/f}$ (n=3 mice) and *Pr-set7*$^{d/d}$ (n=3 mice) uteri on PND5. The values were normalized to *Gapdh* level. Data are presented as mean +/- SEM. Two-tailed unpaired Student's *t*-test. ***p=8e-5 (*Cdkn1a*), *p=0.0329 (*Cdkn2a*). **b** WB analysis of p21 in *Pr-set7*$^{f/f}$ and *Pr-set7*$^{d/d}$ uteri on PND5. GAPDH served as a loading control. **c** IHC analysis of p21 in *Pr-set7*$^{f/f}$ and *Pr-set7*$^{d/d}$ uteri on PND5. Dash lines represent the boundary between the epithelium and the stroma, as well as the stroma and the myometrium. Scale bar: 100μm. **d-f** IHC analysis of Ki67 (**d**), PCNA (**e**) and p-H3 (**f**) in *Pr-set7*$^{f/f}$ and *Pr-set7*$^{d/d}$ uteri on PND5, PND10 and PND15. Dash lines represent the boundary between the epithelium and the stroma, as well as the stroma and the myometrium. Scale bar: 100μm. Source data are provided as a Source Data file.

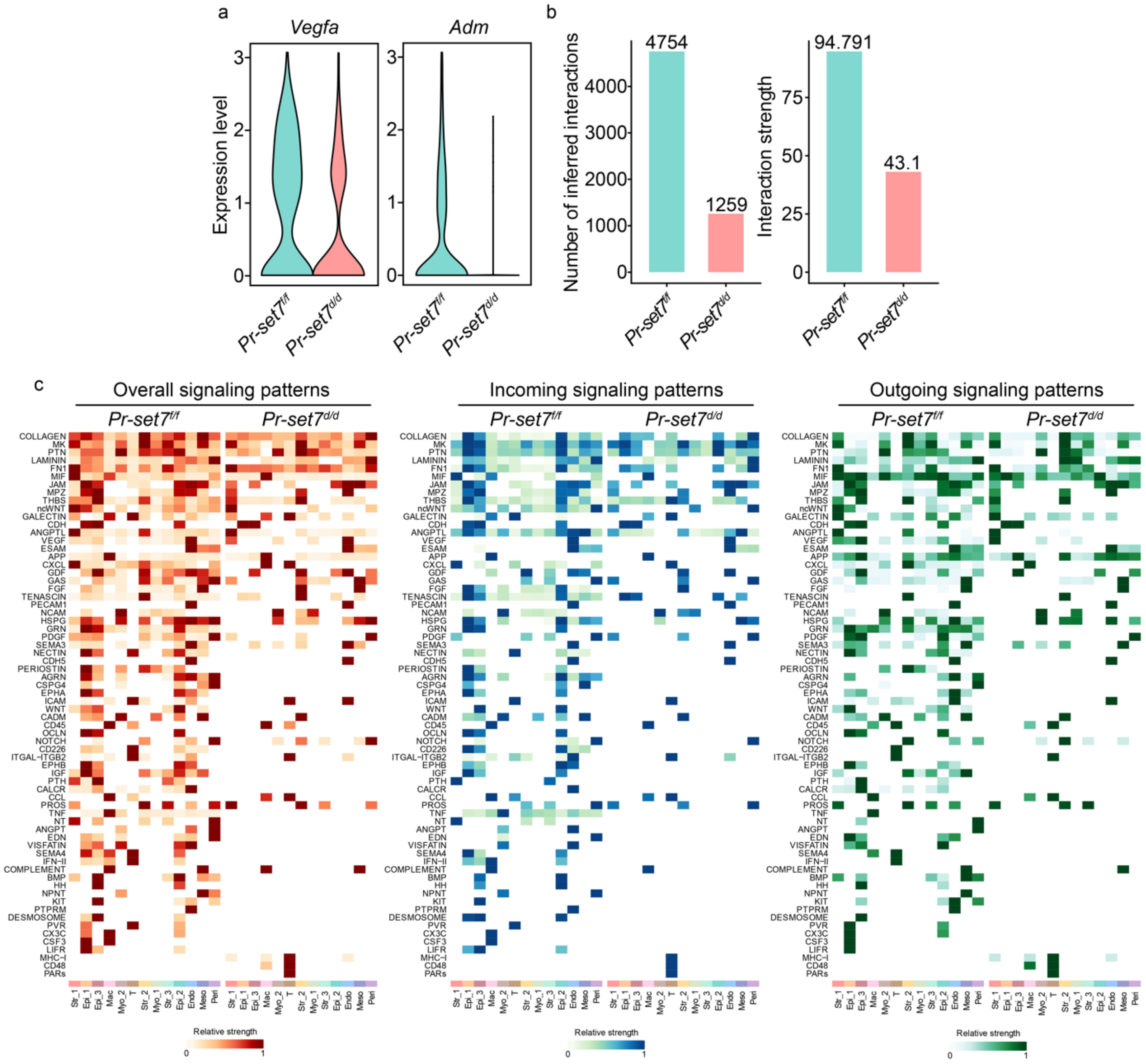

Supplementary Fig. 4 PR-SET7 deficiency hampered inner stromal differentiation and cell-cell interactions.

a Violin plot indicating the expressions of *Vegfa* and *Adm* in the *Pr-set7*$^{f/f}$ and *Pr-set7*$^{d/d}$ inner stroma. **b** CellChat analysis showing the number and strength of cell-cell interactions in the *Pr-set7*$^{f/f}$ and *Pr-set7*$^{d/d}$ uteri. **c** The overall, incoming and outgoing signaling patterns in the *Pr-set7*$^{f/f}$ and *Pr-set7*$^{d/d}$ uteri predicted by CellChat.

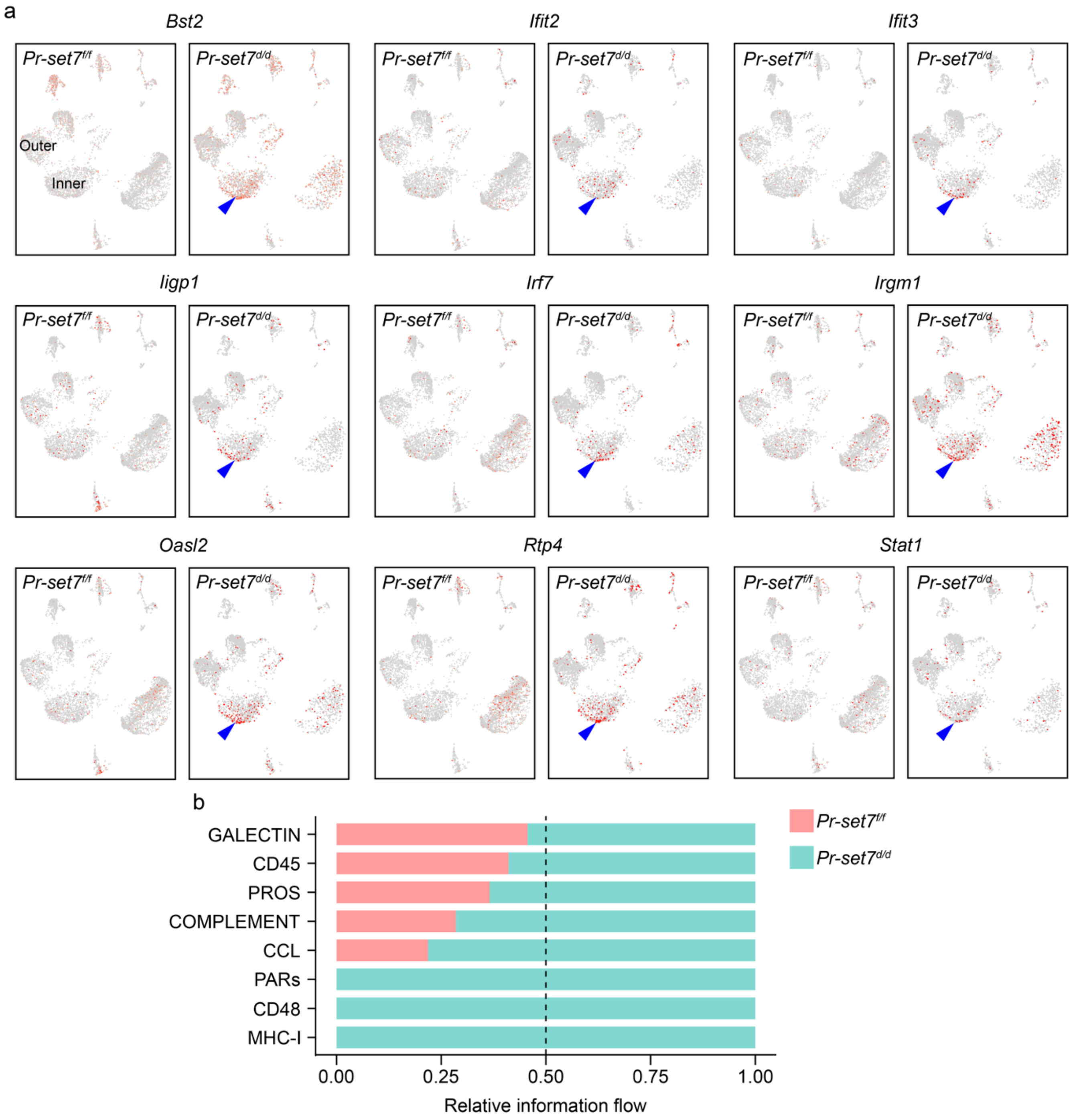

Supplementary Fig. 5 The ablation of *Pr-set7* resulted in hyperactivated innate immune responses in the inner stromal cells.

a UMAP plot showing the expression of ISGs in *Pr-set7*$^{f/f}$ and *Pr-set7*$^{d/d}$ uteri on PND15. Arrowheads indicate the expression of ISGs in the inner stroma of the *Pr-set7*$^{d/d}$ uteri. **b** The relative information flow of immune-related signaling pathways in *Pr-set7*$^{f/f}$ and *Pr-set7*$^{d/d}$ uteri on PND15.

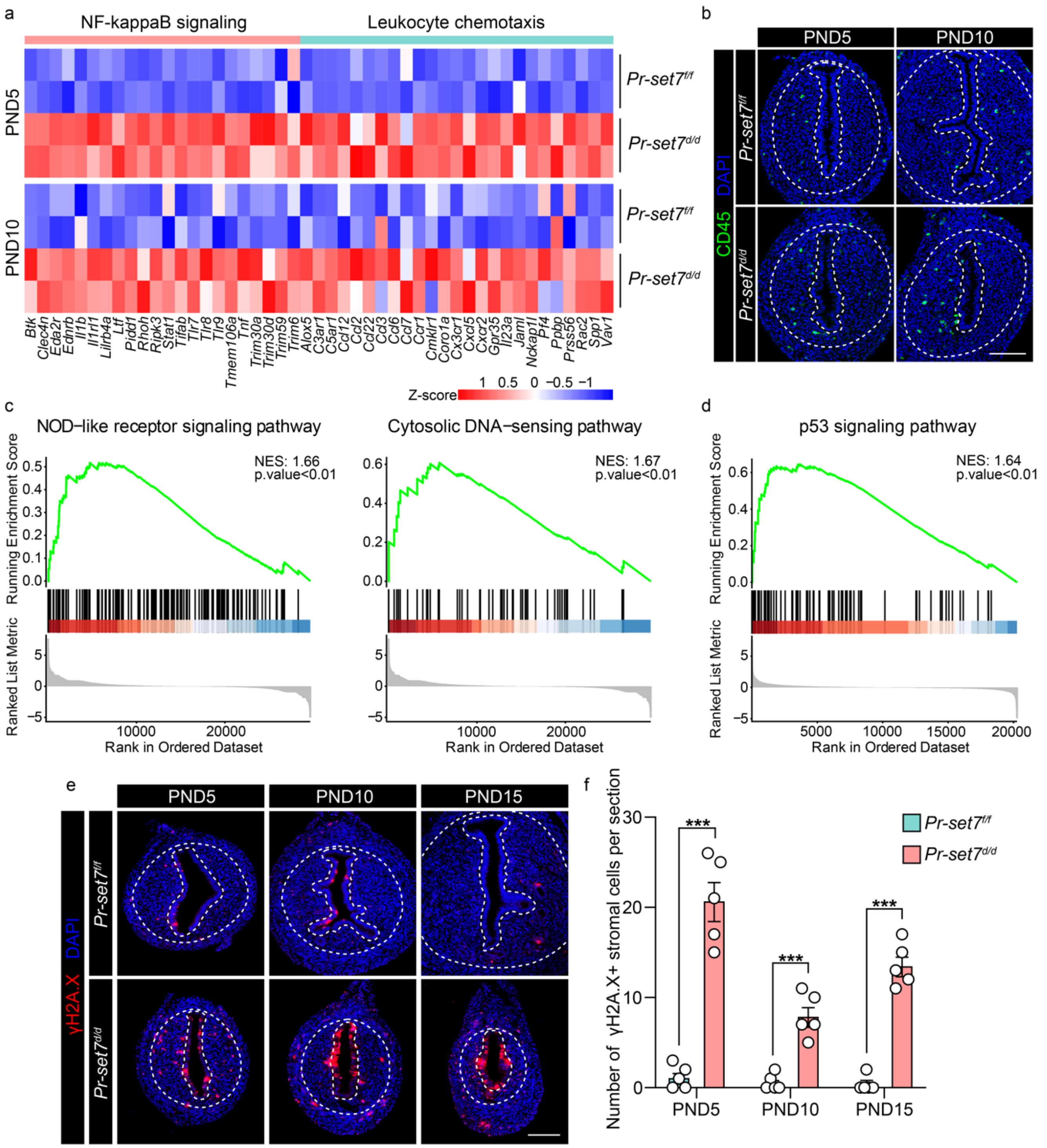
a
NF-kappaB signaling
Leukocyte chemotaxis
PND5
PND10
Pr-set7f/f
Pr-set7d/d
Btk Clec4n Eda2r Ednrb Il1b Il1rl1 Lilrb4a Ltf Pidd1 Rhoh Ripk3 Stat1 Tifab Tlr7 Tlr8 Tlr9 Tmem106a Tnf Trim30a Trim30d Trim59 Trim6 Alox5 C3ar1 C5ar1 Ccl12 Ccl2 Ccl22 Ccl3 Ccl6 Ccl7 Ccr1 Cmklr1 Coro1a Cx3cr1 Cxcl5 Cxcr2 Gpr35 Il23a Jaml Nckap1l Pf4 Ppbp Prss56 Rac2 Spp1 Vav1
Z-score
1 0.5 0 −0.5 −1
b
PND5
PND10
CD45 DAPI
c
NOD−like receptor signaling pathway
NES: 1.66
p.value<0.01
Cytosolic DNA−sensing pathway
NES: 1.67
p.value<0.01
Running Enrichment Score
Ranked List Metric
Rank in Ordered Dataset
d
p53 signaling pathway
NES: 1.64
p.value<0.01
e
PND5
PND10
PND15
γH2A.X DAPI
f
Number of γH2A.X+ stromal cells per section
***
PND5
PND10
PND15

Supplementary Fig. 6 The deletion of *Pr-set7* led to elevated innate immune responses and accumulated DNA damages.

a Heatmap showing the expression of genes associated with the NF-kappaB signaling and leukocyte chemotaxis in *Pr-set7*$^{f/f}$ and *Pr-set7*$^{d/d}$ uteri on PND5 and PND10. **b** IF analysis of CD45 in *Pr-set7*$^{f/f}$ and *Pr-set7*$^{d/d}$ uteri on PND5 and PND10. Dash lines represent the boundary between the epithelium and the stroma, as well as the stroma and the myometrium. Scale bar: 100μm. **c** GSEA analysis indicating the enrichment of NOD-like receptor signaling and cytosolic DNA-sensing pathway related genes in the *Pr-set7*$^{d/d}$ uteri compared with the *Pr-set7*$^{f/f}$ uteri. Significance is based on functional class scoring with clusterProfiler. NES, normalized enrichment score. **d** GSEA analysis indicating the enrichment of p53 signaling pathway related genes in the *Pr-set7*$^{d/d}$ uteri compared with the *Pr-set7*$^{f/f}$ uteri. Significance is based on functional class scoring with clusterProfiler. NES, normalized enrichment score. **e** IF analysis of γH2A.X in *Pr-set7*$^{f/f}$ and *Pr-set7*$^{d/d}$ uteri on PND5, PND10 and PND15. Dash lines represent the boundary between the epithelium and the stroma, as well as the stroma and the myometrium. Scale bar: 100μm. **f** The number of γH2A.X^{+} stromal cells per section in *Pr-set7*$^{f/f}$ (n=5 mice) and *Pr-set7*$^{d/d}$ (n=5 mice) uteri on PND5, PND10 and PND15. Data are presented as mean +/- SEM. Two-tailed unpaired Student's *t*-test. ***p=8e-12 (PND5), ***p=0.0001 (PND10), ***p=2e-8 (PND15). Source data are provided as a Source Data file.

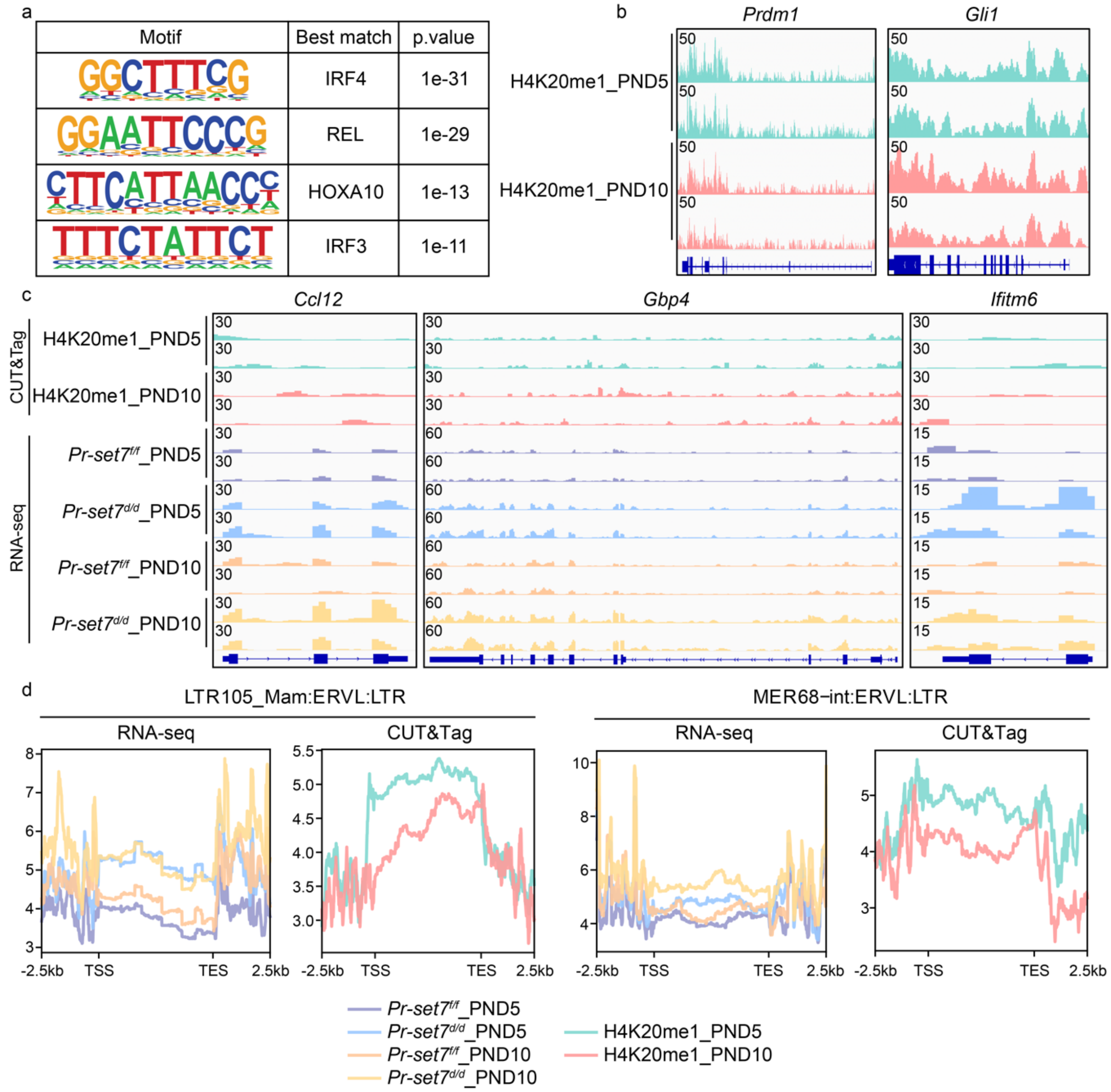
a
Motif
Best match
p.value
IRF4
1e-31
REL
1e-29
HOXA10
1e-13
IRF3
1e-11
b
Prdm1
Gli1
H4K20me1_PND5
H4K20me1_PND10
50
c
Ccl12
Gbp4
Ifitm6
CUT&Tag
RNA-seq
H4K20me1_PND5
H4K20me1_PND10
Pr-set7f/f_PND5
Pr-set7d/d_PND5
Pr-set7f/f_PND10
Pr-set7d/d_PND10
30
60
15
d
LTR105_Mam:ERVL:LTR
MER68-int:ERVL:LTR
RNA-seq
CUT&Tag
-2.5kb
TSS
TES
2.5kb
Pr-set7f/f_PND5
Pr-set7d/d_PND5
Pr-set7f/f_PND10
Pr-set7d/d_PND10
H4K20me1_PND5
H4K20me1_PND10

Supplementary Fig. 7 H4K20me1 regulated the transcription of developmental-related genes and mediated the silencing of ERVs during postnatal uterine development.

a Motif analysis of H4K20me1 CUT&Tag. **b** Genome browser view of normalized H4K20me1 CUT&Tag signals on *Prdm1* and *Gli1*. **c** Genome browser view of normalized H4K20me1 CUT&Tag signals as well as *Pr-set7*$^{f/f}$ and *Pr-set7*$^{d/d}$ RNA-seq signals on *Ccl12*, *Gbp4* and *Ifitm6*. **d** Profile plot showing normalized H4K20me1 CUT&Tag signals as well as *Pr-set7*$^{f/f}$ and *Pr-set7*$^{d/d}$ RNA-seq signals on the body region of LTR105_Mam:ERVL:LTR and MER68-int:ERVL:LTR. TSS, transcription start site; TES, transcription end site.

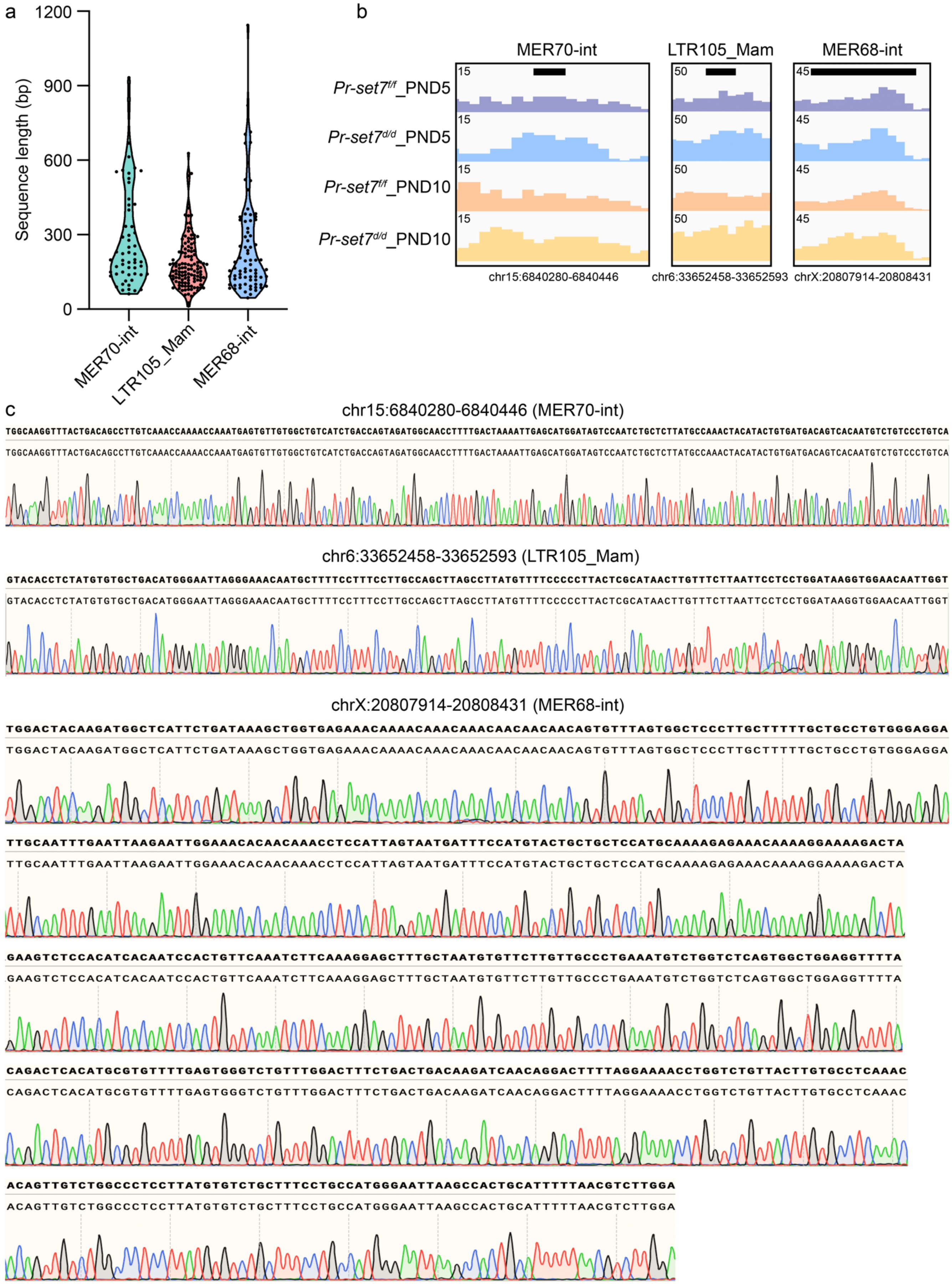
a
Sequence length (bp)
0
300
600
900
1200
MER70-int
LTR105_Mam
MER68-int
b
MER70-int
LTR105_Mam
MER68-int
Pr-set7f/f_PND5
Pr-set7d/d_PND5
Pr-set7f/f_PND10
Pr-set7d/d_PND10
15
50
45
chr15:6840280-6840446
chr6:33652458-33652593
chrX:20807914-20808431
c
chr15:6840280-6840446 (MER70-int)
chr6:33652458-33652593 (LTR105_Mam)
chrX:20807914-20808431 (MER68-int)

Supplementary Fig. 8 The verification of the reads mapping of ERV sequences.

a The sequence lengths of MER70-int, LTR105_Mam and MER68-int. **b** Genome browser view of *Pr-set7*$^{f/f}$ and *Pr-set7*$^{d/d}$ RNA-seq signals on representative ERV copies of MER70-int, LTR105_Mam and MER68-int. **c** The mapping of amplified full-length ERV copies of MER70-int, LTR105_Mam and MER68-int to the sequences downloaded from the UCSC Genome Browser.

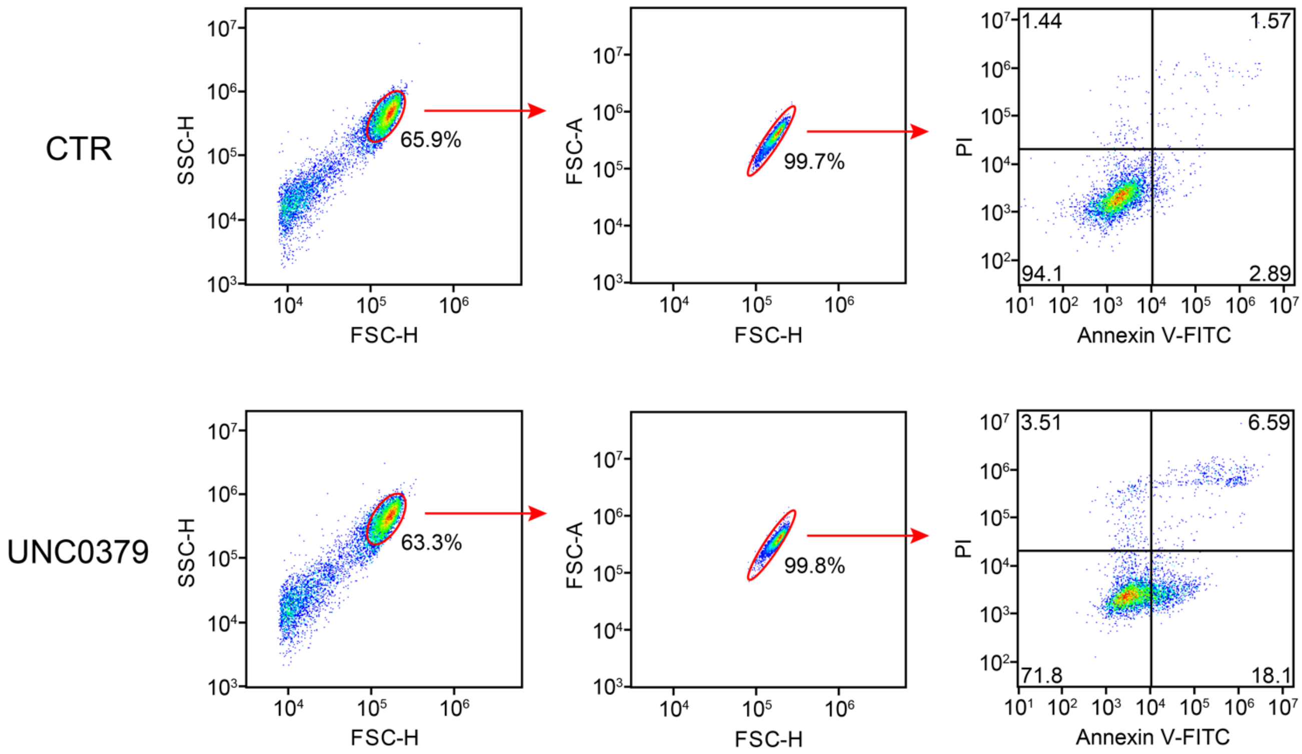

Supplementary Fig. 9 Gating strategy of annexin V-FITC/PI analysis.

Gating strategy of annexin V-FITC/PI analysis of cultured uterine stromal cells treated with DMSO (CTR) or UNC0379. FSC-H and SSC-H were used for initial gating. FSC-H and FSC-A were used to select single cells. Cell death was determined by annexin V-FITC and PI staining: early apoptotic cells ($FITC^+PI^-$), late apoptotic and necrotic cells ($FITC^+PI^+$).

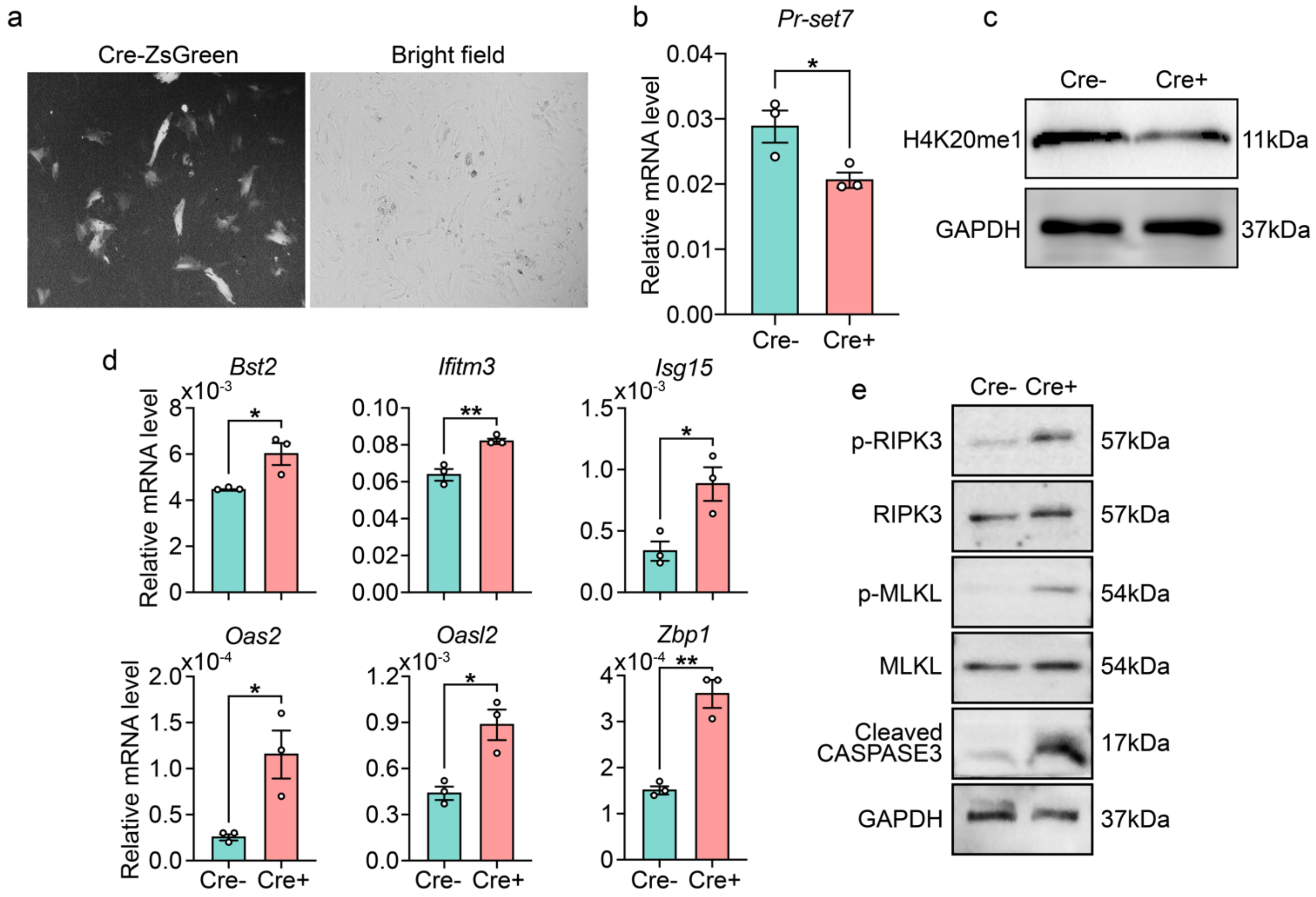
a
Cre-ZsGreen
Bright field
b
Pr-set7
Relative mRNA level
0.04
0.03
0.02
0.01
0.00
*
Cre-
Cre+
c
Cre-
Cre+
H4K20me1
11kDa
GAPDH
37kDa
d
Bst2
Ifitm3
Isg15
Oas2
Oasl2
Zbp1
Relative mRNA level
x10-3
x10-4
*
**
Cre- Cre+
e
Cre- Cre+
p-RIPK3
57kDa
RIPK3
57kDa
p-MLKL
54kDa
MLKL
54kDa
Cleaved CASPASE3
17kDa
GAPDH
37kDa

Supplementary Fig. 10 PR-SET7 deficiency in stromal cells led to viral mimicry responses and cell death.

a Fluorescence showing the efficiency of transient transfection in primary uterine stromal cells. **b** QRT-PCR analysis of *Pr-set7* in *Pr-set7*$^{f/f}$ stromal cells transfected with control (Cre-, n=3 independent biological replicates) or Cre-ZsGreen (Cre+, n=3 independent biological replicates) plasmids. The values were normalized to *Gapdh* level. Data are presented as mean +/- SEM. Two-tailed unpaired Student's *t*-test. *p=0.0399. **c** WB analysis of H4K20me1 in *Pr-set7*$^{f/f}$ stromal cells transfected with control (Cre-) or Cre-ZsGreen (Cre+) plasmids. GAPDH served as a loading control. **d** QRT-PCR analysis of interferon stimulated genes in *Pr-set7*$^{f/f}$ stromal cells transfected with control (Cre-, n=3 independent biological replicates) or Cre-ZsGreen (Cre+, n=3 independent biological replicates) plasmids. The values were normalized to *Gapdh* level. Data are presented as mean +/- SEM. Two-tailed unpaired Student's *t*-test. *p=0.0315 (*Bst2*), **p=0.0064 (*Ifitm3*), *p=0.0258 (*Isg15*), *p=0.0266 (*Oas2*), *p=0.0146 (*Oasl2*), **p=0.0017 (*Zbp1*). **e** WB analysis of necroptosis and apoptosis markers in *Pr-set7*$^{f/f}$ stromal cells transfected with control (Cre-) or Cre-ZsGreen (Cre+) plasmids. GAPDH served as a loading control. Source data are provided as a Source Data file.

Fig. 3c

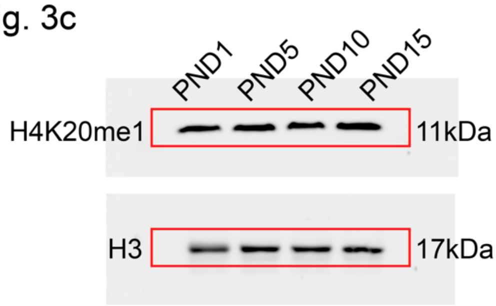

Fig. 3f

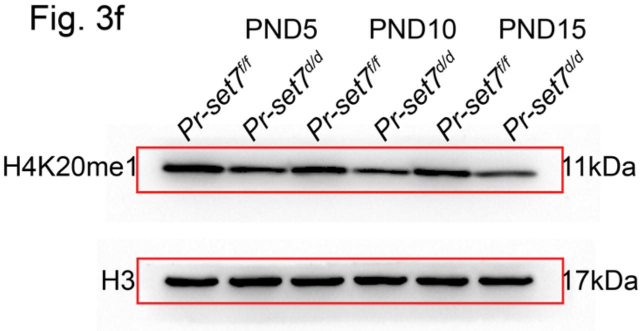

Fig. 5d

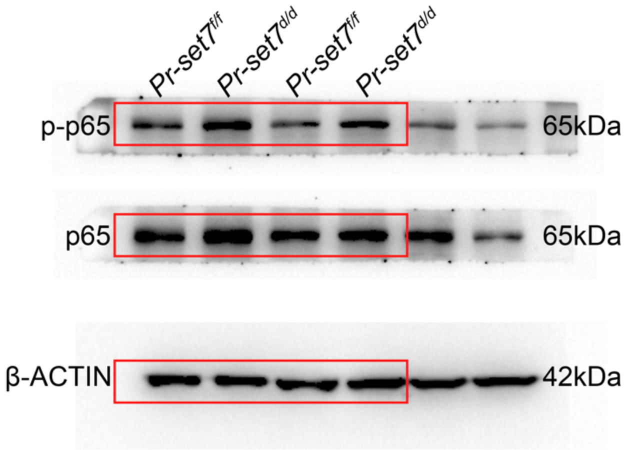

Fig. 5i

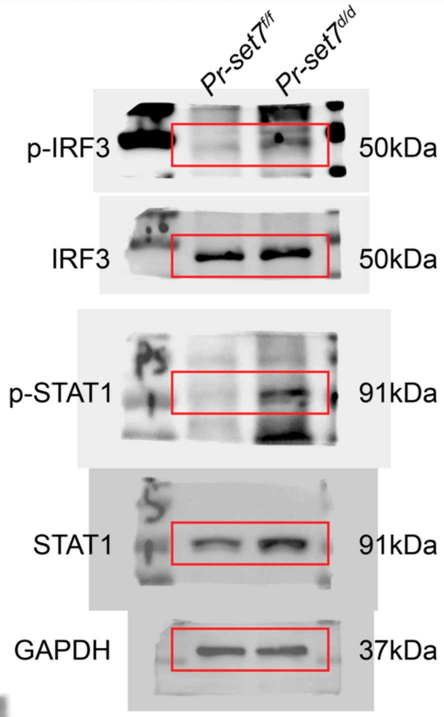

Fig. 7a

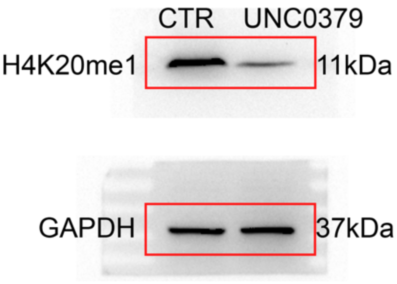

Fig. 7d

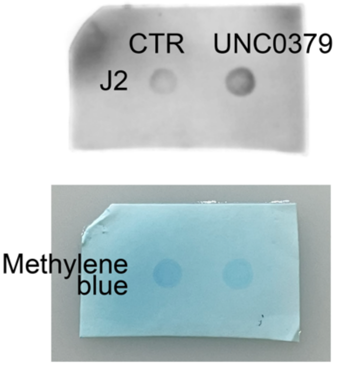

Fig. 7e

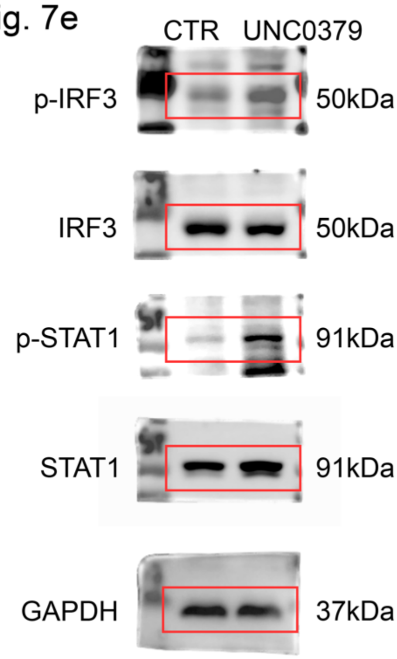

Fig. 7h

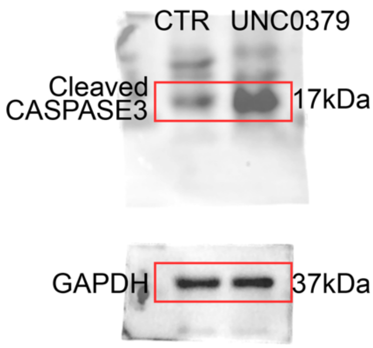

Fig. 7i

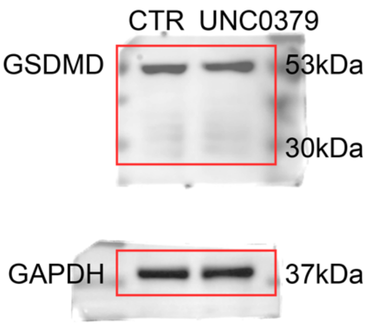

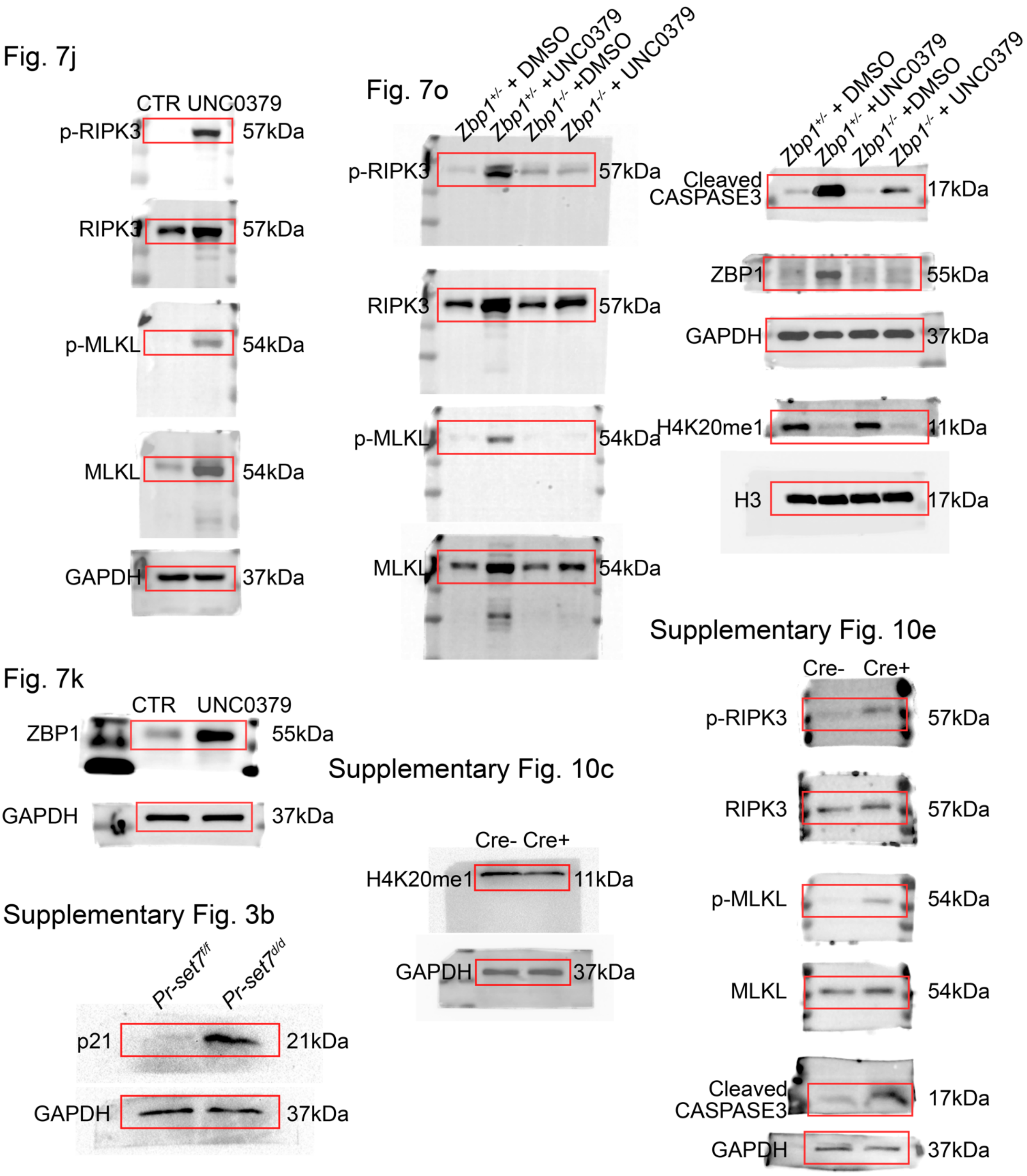

Supplementary Fig. 11 Uncropped blots.

Supplementary Table 1 Information of primers used in this study

**Primers for qRT-PCR**	
**Primer**	**Sequence**
Pr-set7-forward (F)	ATCGCTACCAAGCAGTTCTCC
Pr-set7-reverse (R)	CCCGCTTCTTGGCATCAGTG
Tlr7-F	GGAGCTGGTGGCAAAATTGG
Tlr7-R	TGCTGAGCTGTATGCTCTGG
Tlr8-F	AGTCTGCTCTCTGCACAGCTA
Tlr8-R	GGGGCATGTTTTCCCCTTTC
Tlr9-F	AGCCTCCGAGACAACTACCT
Tlr9-R	TTGGTCAGGGCCTTTAGCTG
Ifitm6-F	CCAGCCGGATCACATTACCTG
Ifitm6-R	GTCCCTGGACTTCACCGAGT
Irf8-F	GACAGGGTCGACAGAGTGTG
Irf8-R	CTCCTGCTTTGACTGAGGGG
Isg15-F	TGGTACAGAACTGCAGCGAG
Isg15-R	AGCCAGAACTGGTCTTCGTG
Oas2-F	GAGTGGGAGGTGACGTTTGA
Oas2-R	GATCCTTGTAGGCTTCGGGC
Zbp1-F	GGCTCTGGGAATGACGACAG
Zbp1-R	CCTTCCTGACGTGAGTGGTA
MER70-int-F	TGGCAAGGTTTACTGACAGC
MER70-int-R	TGACAGGGACAGACATTGTG
LTR105_Mam-F	GTACACCTCTATGTGTGCTGAC
LTR105_Mam-R	ACCAATTGTTCCACCTTATCCA
MER68-int-F	GTTTGCATTGCTCTCTTCTCC
MER68-int-R	GCTCTAGTAAGGAAACTTGGTT
Bst2-F	CACAGGCAAACTCCTGCAAC

Bst2-R	TCCTGGTTCAGCTTCGTGAC
Ifitm3-F	AGCCTATGCCTACTCCGTGA
Ifitm3-R	AGTGTGAAGGTTTTGAGCGT
Oasl2-F	CTGCAGTGCCTGAGACGTAA
Oasl2-R	AGATGCAGATGTCGCGGTAG
Ccnd1-F	AGCCCTTCTGGAGTCAAGCC
Ccnd1-R	CACATCTCGCACGTCGGTG
Ccnd2-F	AAGGAGGTAAGGGAAGCACTC
Ccnd2-R	CCTCGATGGTCAACAGGTTCT
Ccnd3-F	CCTCCTACTTCCAGTGCGTG
Ccnd3-R	GGCAGACGGTACCTAGAAGC
Ccne1-F	CACAGCTTCGGGTCTGAGTT
Ccne1-R	TGCAAAAACACGGCCACATT
Ccna1-F	GGAAATTGCAGCTTGTCGGG
Ccna1-R	GGTGGTTGGAACGGTCAGAT
Ccnb1-F	TAAGGCCGTGACAAAGGCAT
Ccnb1-R	TCGACAACTTCCGTTAGCCT
Cdkn1a-F	CAGGCACCATGTCCAATCCT
Cdkn1a-R	AAAGTTCCACCGTTCTCGGG
Cdkn2a-F	AGGACCCCACTACCTTCTCC
Cdkn2a-R	CCAGCGGAACGCAAATATCG
**Primers for SCRINSHOT**	
**Primer**	**Sequence**
Wnt16-1	AGAAAGAAATCGTAGACTAGTCCTCTATGATTACT GACTGCGTCTATTTAGTGGAGCCGCCCCTATCTT CTTTATTTGAAGCCTGCCAGAA
Wnt16-2	TACAGAGGCGTCTCATGCTATCCTCTATGATTACT GACTGCGTCTATTTAGTGGAGCCGCCCCTATCTT CTTTCAACACCACACCTTGGTTAC

Htra3-1	TGATGGCATCGGTCTGGATTCCTCTATGATTACTGACTGCGTCTATTTAGTGGAGCCGCCCCTATCTTCTTTTCCTGAGTTCCCGTAATTGA
Htra3-2	CCTGGATGGTGGCCTCATTCCTCTATGATTACTGACTGCGTCTATTTAGTGGAGCCGCCCCTATCTTCTTTGTCCGACTTCTTGTCGATGT
Tlr7-1	AAGACTCTAGAAACTAAGAGTCCTCTATGATTACTGACTGCGTCTATTTAGTGGAGCCGCCCCTATCTTCTTTGGAAACCATCGAAACCCA
Tlr7-2	GCATTTGAGGAATGAAAGATTCCTCTATGATTACTGACTGCGTCTATTTAGTGGAGCCGCCCCTATCTTCTTTTGTTTCCTGATAAGTTGAG
Tlr7-3	TGATGGAGAAGATGTTGTTATCCTCTATGATTACTGACTGCGTCTATTTAGTGGAGCCGCCCCTATCTTCTTTTCTGTTAGATTCTCCTTCG
Isg15-1	TGCGTCAGAAAGACCTCATATCCTCTATGATTACTGACTGCGTCTATTTAGTGGAGCCGCCCCTATCTTCTTTTCTTAAGCGTGTCTACAGTC
Isg15-2	AGAAAGACCTCATAGATGTTTCCTCTATGATTACTGACTGCGTCTATTTAGTGGAGCCGCCCCTATCTTCTTTTGTCTACAGTCTGCGTC
Zbp1-1	TCATCAAGGCTAGGCTGTCCTCTATGATTACTGACTGCGTCTATTTAGTGGAGCCGCCCCTATCTTCTTTTTCCAGGAATCTTAATATTCTT
Zbp1-2	TTATTTCTCATGGAATACAGGTCCTCTATGATTACTGACTGCGTCTATTTAGTGGAGCCGCCCCTATCTTCTTTTCATAGCTCAGAAGGTGC

Zbp1-3	CGGTAAAGGACTTGATTGAGTCCTCTATGATTACT GACTGCGTCTATTTAGTGGAGCCGCCCCTATCTT CTTTCTGTCCTCCTTCTTCAGG
**Primers for ISH**	
**Primer**	**Sequence**
Pr-set7-ISH-F	AATTAACCCTCACTAAAGGGTCGCTAAGCAAGCC
Pr-set7-ISH-R	TAATACGACTCACTATAGGGATGAGTCTCCCTAG
**Primers for ChIP-qRT-PCR**	
**Primer**	**Sequence**
Zbp1-ChIP-F	ACTCAGACCGGAAGTTGGTG
Zbp1-ChIP-R	ACCACAAAGCCAAGATCGGT

Supplementary Table 2 Information of antibodies used in this study

Antibodies for immunoblotting		
**Antibody**	**Company**	**Dilution**
H4K20me1	ABclonal (A2370)	1:1000
H3	Abmart (P30266)	1:5000
p65	CST (8242)	1:1000
p-p65	CST (3033)	1:1000
β-ACTIN	Bioworld (AP0060)	1:5000
IRF3	CST (4302)	1:1000
p-IRF3	CST (79945)	1:1000
STAT1	CST (9172)	1:1000
p-STAT1	CST (9167)	1:1000
GAPDH	Bioworld (AP0063)	1:5000
J2	SCICONS (10010500)	1:5000
RIPK3	CST (15828)	1:1000
p-RIPK3	CST (91702)	1:1000
MLKL	CST (37705)	1:1000
p-MLKL	CST (37333)	1:1000
Cleaved CASPASE-3	CST (9661)	1:1000
GSDMD	Abcam (ab219800)	1:1000
ZBP1	Santa Cruz (sc-271483)	1:500
p21	ABclonal (A19094)	1:1000
**Antibodies for immunostaining**		
**Antibody**	**Company**	**Dilution**
PR	CST (8757)	1:200
H4K20me1	ABclonal (A2370)	1:200
WT1	Santa Cruz (sc-393498)	1:200
VIMENTIN	Abcam (ab92547)	1:200

α-SMA	BioGenex (MU128-UC)	1:200
PDGFRα	CST (3174)	1:200
F4/80	CST (70076)	1:200
J2	SCICONS (10010500)	1:500
p21	ABclonal (A19094)	1:200
Ki67	Abcam (ab15580)	1:100
PCNA	Santa Cruz (sc-7907)	1:200
p-H3	CST (9701)	1:500
CD45	CST (70257)	1:200
γH2A.X	CST (9718)	1:500
**Antibodies for CUT&Tag and ChIP-qRT-PCR**		
**Antibody**	**Company**	
H4K20me1	ActiveMotif (39727)	
