# Supplementary material for: PR-SET7 epigenetically restrains uterine interferon response and cell death governing proper postnatal stromal development
Source: Nat Commun. 2024 Jun 10;15:4920. doi: 10.1038/s41467-024-49342-6 (PMC11164956; doi:10.1038/s41467-024-49342-6)
Supplement: Supplementary file 3 — Reporting Summary [file 41467_2024_49342_MOESM3_ESM.pdf]

nature portfolio

Corresponding author(s): Haibin Wang, Wenbo Deng, Shuangbo Kong

Last updated by author(s): May 20, 2024

Reporting Summary

Nature Portfolio wishes to improve the reproducibility of the work that we publish. This form provides structure for consistency and transparency in reporting. For further information on Nature Portfolio policies, see our Editorial Policies and the Editorial Policy Checklist.

Statistics

For all statistical analyses, confirm that the following items are present in the figure legend, table legend, main text, or Methods section.

n/a	Confirmed	
☐	☒	The exact sample size (n) for each experimental group/condition, given as a discrete number and unit of measurement
☐	☒	A statement on whether measurements were taken from distinct samples or whether the same sample was measured repeatedly
☐	☒	The statistical test(s) used AND whether they are one- or two-sided<br>*Only common tests should be described solely by name; describe more complex techniques in the Methods section.*
☒	☐	A description of all covariates tested
☐	☒	A description of any assumptions or corrections, such as tests of normality and adjustment for multiple comparisons
☐	☒	A full description of the statistical parameters including central tendency (e.g. means) or other basic estimates (e.g. regression coefficient) AND variation (e.g. standard deviation) or associated estimates of uncertainty (e.g. confidence intervals)
☐	☒	For null hypothesis testing, the test statistic (e.g. F, t, r) with confidence intervals, effect sizes, degrees of freedom and P value noted<br>*Give P values as exact values whenever suitable.*
☒	☐	For Bayesian analysis, information on the choice of priors and Markov chain Monte Carlo settings
☒	☐	For hierarchical and complex designs, identification of the appropriate level for tests and full reporting of outcomes
☒	☐	Estimates of effect sizes (e.g. Cohen's d, Pearson's r), indicating how they were calculated

Our web collection on statistics for biologists contains articles on many of the points above.

Software and code

Policy information about availability of computer code

Data collection	Zen 2.3 (Zeiss) and NIS-Elements D 4.50.00 (Nikon) were used for image capturing. QuantStudio Design&Analysis Software was used for qRT-PCR data collection. Image Lab Software was used for WB data collection. CytExpert 2.4 was used for flow cytometry data collection.

Data analysis	GraphPad Prism (8.2.0)<br>FlowJo (10.8.1)<br>Trim Galore (0.6.4)<br>STAR (2.7.3)<br>DESeq2 (1.30.1)<br>ggplot2 (3.4.3)<br>pheatmap (1.0.12)<br>clusterProfiler (4.8.1)<br>Bowtie2 (2.5.0)<br>MACS2 (2.2.7.1)<br>deepTools (3.5.1)<br>ChIPseeker (1.14.2)<br>IGV (2.11.6)<br>Seurat (4.3.0)<br>Cellchat (1.5.0)

For manuscripts utilizing custom algorithms or software that are central to the research but not yet described in published literature, software must be made available to editors and reviewers. We strongly encourage code deposition in a community repository (e.g. GitHub). See the Nature Portfolio guidelines for submitting code & software for further information.

Data

Policy information about availability of data

All manuscripts must include a data availability statement. This statement should provide the following information, where applicable:

- Accession codes, unique identifiers, or web links for publicly available datasets
- A description of any restrictions on data availability
- For clinical datasets or third party data, please ensure that the statement adheres to our policy

The single-cell RNA-seq, bulk RNA-seq and CUT&Tag data generated in this study have been deposited in the National Center for Biotechnology Information Sequence Read Archive under accession code PRJNA1046685 [https://www.ncbi.nlm.nih.gov/bioproject/PRJNA1046685].
Source data are provided with this paper.
Codes used in this study have been deposited at [https://github.com/dwb0211/PRSet7-scRNA].

Research involving human participants, their data, or biological material

Policy information about studies with human participants or human data. See also policy information about sex, gender (identity/presentation), and sexual orientation and race, ethnicity and racism.

Reporting on sex and gender	*Use the terms sex (biological attribute) and gender (shaped by social and cultural circumstances) carefully in order to avoid confusing both terms. Indicate if findings apply to only one sex or gender; describe whether sex and gender were considered in study design; whether sex and/or gender was determined based on self-reporting or assigned and methods used.*<br>*Provide in the source data disaggregated sex and gender data, where this information has been collected, and if consent has been obtained for sharing of individual-level data; provide overall numbers in this Reporting Summary. Please state if this information has not been collected.*<br>*Report sex- and gender-based analyses where performed, justify reasons for lack of sex- and gender-based analysis.*
Reporting on race, ethnicity, or other socially relevant groupings	*Please specify the socially constructed or socially relevant categorization variable(s) used in your manuscript and explain why they were used. Please note that such variables should not be used as proxies for other socially constructed/relevant variables (for example, race or ethnicity should not be used as a proxy for socioeconomic status).*<br>*Provide clear definitions of the relevant terms used, how they were provided (by the participants/respondents, the researchers, or third parties), and the method(s) used to classify people into the different categories (e.g. self-report, census or administrative data, social media data, etc.)*<br>*Please provide details about how you controlled for confounding variables in your analyses.*
Population characteristics	*Describe the covariate-relevant population characteristics of the human research participants (e.g. age, genotypic information, past and current diagnosis and treatment categories). If you filled out the behavioural & social sciences study design questions and have nothing to add here, write "See above."*
Recruitment	*Describe how participants were recruited. Outline any potential self-selection bias or other biases that may be present and how these are likely to impact results.*
Ethics oversight	*Identify the organization(s) that approved the study protocol.*

Note that full information on the approval of the study protocol must also be provided in the manuscript.

Field-specific reporting

Life sciences study design

All studies must disclose on these points even when the disclosure is negative.

Sample size	Sample size was determined to reflect biological and technical variance of the investigated parameters based on previouly published literature (PMID: 35194044). For all immunostaining, in situ hybridization, WB, qRT-PCR and flow cytometry experiments, at least three independent biological replicates were used. For RNA-seq and CUT&Tag, two independent biological replicates were used.
Data exclusions	No data were excluded from the analyses.
Replication	For all immunostaining, in situ hybridization, WB, qRT-PCR and flow cytometry experiments, at least three independent biological replicates were used. For RNA-seq and CUT&Tag, two independent biological replicates were used.
Randomization	All samples were allocated at random.
Blinding	The investigators were blinded to group allocation during data collection/analysis.

Reporting for specific materials, systems and methods

We require information from authors about some types of materials, experimental systems and methods used in many studies. Here, indicate whether each material, system or method listed is relevant to your study. If you are not sure if a list item applies to your research, read the appropriate section before selecting a response.

Materials & experimental systems

n/a	Involved in the study	
☐	☒	Antibodies
☒	☐	Eukaryotic cell lines
☒	☐	Palaeontology and archaeology
☐	☒	Animals and other organisms
☒	☐	Clinical data
☒	☐	Dual use research of concern
☒	☐	Plants

Methods

n/a	Involved in the study	
☐	☒	ChIP-seq
☐	☒	Flow cytometry
☒	☐	MRI-based neuroimaging

Antibodies

Antibodies used	For immunostaining, PR (CST, 8757), H4K20me1 (ABclonal, A2370), WT1 (Santa Cruz, sc-393498), VIMENTIN (Abcam, ab92547), α-SMA (BioGenex, MU128-UC), PDGFRα (CST, 3174), F4/80 (CST, 70076), J2 (SCICONS, 10010500), p21 (ABclonal, A19094), Ki67 (Abcam, ab15580), PCNA (Santa Cruz, sc-7907), p-H3 (CST, 9701), CD45 (CST, 70257) and γH2A.X (CST, 9718).<br>For immunoblotting, H4K20me1 (ABclonal, A2370), H3 (Abmart, P30266), p65 (CST, 8242), p-p65 (CST, 3033), β-ACTIN (Bioworld, AP0060), IRF3 (CST, 4302), p-IRF3 (CST, 79945), STAT1 (CST, 9172), p-STAT1 (CST, 9167), GAPDH (Bioworld, AP0063), J2 (SCICONS, 10010500), RIPK3 (CST, 15828), p-RIPK3 (CST, 91702), MLKL (CST, 37705), p-MLKL (CST, 37333), Cleaved CASPASE-3 (CST, 9661), GSDMD (Abcam, ab219800), ZBP1 (Santa Cruz, sc-271483) and p21 (ABclonal, A19094) .<br>For CUT&Tag, H4K20me1 (ActiveMotif, 39727).
Validation	Validation of the commercial antibodies were done by the manufacturers.<br>Below shows the relevant information listed on the suppliers' websites for antibodies used in this article:<br>PR (CST, 8757), https://www.cellsignal.cn/products/primary-antibodies/progesterone-receptor-a-b-d8q2j-xp-174-rabbit-mab/8757<br>H4K20me1 (ABclonal, A2370), https://abclonal.com.cn/catalog/A2370<br>WT1 (Santa Cruz, sc-393498), https://www.scbt.com/zh/p/wt1-antibody-h-1<br>VIMENTIN (Abcam, ab92547), https://www.abcam.com/products/primary-antibodies/vimentin-antibody-epr3776-cytoskeleton-marker-ab92547.html<br>α-SMA (BioGenex, MU128-UC), https://biogenex.com/product/anti-actin-smooth-muscle/<br>PDGFRα (CST, 3174), https://www.cellsignal.cn/products/primary-antibodies/pdgf-receptor-a-d1e1e-xp-rabbit-mab/3174<br>F4/80 (CST, 70076), https://www.cellsignal.cn/products/primary-antibodies/f4-80-d2s9r-xp-174-rabbit-mab/70076<br>J2 (SCICONS, 10010500), https://www.nordicmubio.com/products/scicons-anti-dsrna-antibody-j2/10010500<br>p21 (ABclonal, A19094), https://abclonal.com.cn/catalog/A19094<br>Ki67 (Abcam, ab15580), https://www.abcam.cn/products/primary-antibodies/ki67-antibody-ab15580.html<br>PCNA (Santa Cruz, sc-7907), https://www.scbt.com/zh/p/pcna-antibody-fl-261<br>p-H3 (CST, 9701), https://www.cellsignal.cn/products/primary-antibodies/phospho-histone-h3-ser10-antibody/9701<br>CD45 (CST, 70257), https://www.cellsignal.cn/products/primary-antibodies/cd45-d3f8q-rabbit-mab/70257<br>γH2A.X (CST, 9718), https://www.cellsignal.cn/products/primary-antibodies/phospho-histone-h2a-x-ser139-20e3-rabbit-mab/9718<br>H3 (Abmart, P30266), http://www.ab-mart.com.cn/page.aspx?node=%2059%20&id=%20996<br>p65 (CST, 8242), https://www.cellsignal.cn/products/primary-antibodies/nf-kb-p65-d14e12-xp-174-rabbit-mab/8242

p-p65 (CST, 3033), https://www.cellsignal.cn/products/primary-antibodies/phospho-nf-kb-p65-ser536-93h1-rabbit-mab/3033
β-ACTIN (Bioworld, AP0060), https://www.bioworlde.com/Primary-Antibodies/127218.html
IRF3 (CST, 4302), https://www.cellsignal.cn/products/primary-antibodies/irf-3-d83b9-rabbit-mab/4302
p-IRF3 (CST, 79945), https://www.cellsignal.cn/products/primary-antibodies/phospho-irf-3-ser379-e6f7q-rabbit-mab/79945
STAT1 (CST, 9172), https://www.cellsignal.cn/products/primary-antibodies/stat1-antibody/9172
p-STAT1 (CST, 9167), https://www.cellsignal.cn/products/primary-antibodies/phospho-stat1-tyr701-58d6-rabbit-mab/9167
GAPDH (Bioworld, AP0063), https://www.bioworlde.com/Primary-Antibodies/127232.html
RIPK3 (CST, 15828), https://www.cellsignal.cn/products/primary-antibodies/rip3-d8j3l-rabbit-mab/15828
p-RIPK3 (CST, 91702), https://www.cellsignal.cn/products/primary-antibodies/phospho-rip3-thr231-ser232-e7s1r-rabbit-mab/91702
MLKL (CST, 37705), https://www.cellsignal.cn/products/primary-antibodies/mlkl-d6w1k-rabbit-mab/37705
p-MLKL (CST, 37333), https://www.cellsignal.cn/products/primary-antibodies/phospho-mlkl-ser345-d6e3g-rabbit-mab/37333
Cleaved CASPASE-3 (CST, 9661), https://www.cellsignal.cn/products/primary-antibodies/cleaved-caspase-3-asp175-antibody/9661
GSDMD (Abcam, ab219800), https://www.abcam.cn/products/primary-antibodies/gsdmd-antibody-epr20859-ab219800.html
ZBP1 (Santa Cruz, sc-271483), https://www.scbt.com/zh/p/zbp1-antibody-h-9
H4K20me1 (ActiveMotif, 39727), https://www.activemotif.com/catalog/details/39727/histone-h4-monomethyl-lys20-antibody-mab-clone-5e10-d8

Animals and other research organisms

Policy information about studies involving animals; ARRIVE guidelines recommended for reporting animal research, and Sex and Gender in Research

Laboratory animals	All Pr-set7 f/f, Pr-set7 d/d and Zbp1-/- mice used in this study were 1-15 days old and were C57BL6 strains.
Wild animals	The study did not involve wild animals.
Reporting on sex	Uterine samples were collected from female neonates.
Field-collected samples	The study did not involve samples collected from the field.
Ethics oversight	All mice were housed in the animal care facility of Xiamen University (temperature: 22±2°C, humidity: 50-60%, 12h light/dark cycle) according to the guidelines for the care and use of laboratory animals. All experimental procedures were approved by the Animal Care Committee of Xiamen University (XMULAC20170366).

Note that full information on the approval of the study protocol must also be provided in the manuscript.

Plants

Seed stocks	*Report on the source of all seed stocks or other plant material used. If applicable, state the seed stock centre and catalogue number. If plant specimens were collected from the field, describe the collection location, date and sampling procedures.*
Novel plant genotypes	*Describe the methods by which all novel plant genotypes were produced. This includes those generated by transgenic approaches, gene editing, chemical/radiation-based mutagenesis and hybridization. For transgenic lines, describe the transformation method, the number of independent lines analyzed and the generation upon which experiments were performed. For gene-edited lines, describe the editor used, the endogenous sequence targeted for editing, the targeting guide RNA sequence (if applicable) and how the editor was applied.*
Authentication	*Describe any authentication procedures for each seed stock used or novel genotype generated. Describe any experiments used to assess the effect of a mutation and, where applicable, how potential secondary effects (e.g. second site T-DNA insertions, mosiacism, off-target gene editing) were examined.*

ChIP-seq

Data deposition

☒ Confirm that both raw and final processed data have been deposited in a public database such as GEO.

☒ Confirm that you have deposited or provided access to graph files (e.g. BED files) for the called peaks.

Data access links<br>*May remain private before publication.*	https://www.ncbi.nlm.nih.gov/bioproject/PRJNA1046685
Files in database submission	PND5_H4K20_Cut_Tag_1<br>PND5_H4K20_Cut_Tag_2<br>PND10_H4K20_Cut_Tag_1<br>PND10_H4K20_Cut_Tag_2
Genome browser session<br>(e.g. UCSC)	IGV 2.8.13

Methodology

Replicates	Each experiment has two biological replicates.
Sequencing depth	PND5_H4K20_Cut_Tag_1: paired-end; read length:300; total reads:31789891; Uniquely mapped reads:27282893<br>PND5_H4K20_Cut_Tag_2: paired-end; read length:300; total reads:31651459; Uniquely mapped reads:27586137<br>PND10_H4K20_Cut_Tag_1: paired-end; read length:300; total reads:27321670; Uniquely mapped reads:24164939<br>PND10_H4K20_Cut_Tag_2: paired-end; read length:300; total reads:21087973; Uniquely mapped reads:18645735
Antibodies	H4K20me1 (ActiveMotif, 39727)
Peak calling parameters	macs2 command line: callpeak -t H4K20-P10-DAligned.sortedByCoord.out.bam -f BAM -g mm -n H4K20-P10-D --nomodel -B
Data quality	PND5_H4K20_Cut_Tag_1: 17315 peaks<br>PND5_H4K20_Cut_Tag_2: 16652 peaks<br>PND10_H4K20_Cut_Tag_1: 15187 peaks<br>PND10_H4K20_Cut_Tag_2: 11034 peaks
Software	Bowtie2: https://bowtie-bio.sourceforge.net/bowtie2/index.shtml<br>MACS2: https://github.com/macs3-project/MACS

Flow Cytometry

Plots

Confirm that:

- [x] The axis labels state the marker and fluorochrome used (e.g. CD4-FITC).
- [x] The axis scales are clearly visible. Include numbers along axes only for bottom left plot of group (a 'group' is an analysis of identical markers).
- [x] All plots are contour plots with outliers or pseudocolor plots.
- [x] A numerical value for number of cells or percentage (with statistics) is provided.

Methodology

Sample preparation	Cultured mouse endometrial stromal cells treated with DMSO *or* UNC0379 were harvested and stained with Annexin V-FITC and propidium iodide (PI) in the dark for 20min at room temperature according to manufacturer's instructions of the Annexin V-FITC Apoptosis Detection Kit (Beyotime, C1062). After staining, cells were immediately examined by flow cytometry.
Instrument	Flow cytometry was performed using Beckman CytoFLEX S.
Software	Flow cytometry data were collected by the CytExpert 2.4 software and analyzed by the FlowJo_v10 software.
Cell population abundance	*Describe the abundance of the relevant cell populations within post-sort fractions, providing details on the purity of the samples and how it was determined.*
Gating strategy	FSC-H and SSC-H were used for initial gating. FSC-H and FSC-A were used to select single cells. Cell death was determined by Annexin V-FITC and PI staining: early apoptotic cells (FITC+PI-), late apoptotic and necrotic cells (FITC+PI+). Unstained cells were used as negative control.

- [x] Tick this box to confirm that a figure exemplifying the gating strategy is provided in the Supplementary Information.
